# Electrodermal activity – a promising biomarker for cardiovascular risk assessment in adolescent anorexia nervosa

**DOI:** 10.1192/j.eurpsy.2021.547

**Published:** 2021-08-13

**Authors:** I. Tonhajzerova, N. Ferencova, I. Ondrejka, L. Bona Olexova, D. Funakova, I. Hrtanek, Z. Visnovcova

**Affiliations:** 1 Department Of Physiology, Jessenius Faculty of Medicine Comenius University, Martin, Slovak Republic; 2 Psychiatric Clinic, Jessenius Faculty of Medicine in MartinComenius University in Bratislava, University Hospital Martin, Martin, Slovak Republic; 3 Biomedical Center Martin, Jessenius Faculty of Medicine in MartinComenius University in Bratislava, Martin, Slovak Republic

**Keywords:** anorexia nervosa, electrodermal activity, sympathetic nervous system

## Abstract

**Introduction:**

Anorexia nervosa (AN) represents a severe mental disorder associated with cardiovascular complications leading to morbidity and mortality. Abnormal functioning of autonomic nervous system, particularly sympathetic nervous system, plays a crucial role in AN-linked psychopathology and cardiovascular diseases; however, the pathomechanisms are still unclear.

**Objectives:**

Thus, we studied sympathetic arousal in response to mental stress using conventional parameters, and for the first time by spectral analysis of electrodermal activity with aim to detect non-invasive biomarkers for cardiovascular risk assessment already in adolescent AN patients.

**Methods:**

Twenty-five AN girls were examined (14.8±0.4 yr.) and age/gender matched controls (15.1±0.3 years). Electrodermal activity (EDA) was continuously recorded at rest (5 min.) and in response to Go/NoGo test (5 min.). Evaluated parameters: skin conductance level (SCL) and spectral parameter of EDA in the sympathetic frequency band (EDASymp). EDA reactivity was calculated as percentual change (%) of SCL and EDASymp in response to stressor.

**Results:**

The AN group had significantly reduced SCL and EDASymp compared to controls during baseline (p=0.041, p=0.0001, respectively) and in response to Go/NoGo test (p=0.043, p=0.017, respectively). The EDASymp index reactivity was significantly lower in AN group compared to control (p=0.034).

**Conclusions:**

Our study revealed resting sympathetic underactivity associated with lower reactivity to mental stressor indexed by EDA parameters in adolescent AN patients. This altered pattern of sympathetic arousal could play important role as a pathomechanism leading to cardiovascular complications in AN. It seems that EDA indices represent potential non-invasive biomarkers to detect AN-linked cardiovascular risk already at adolescent age.

**Conflict of interest:**

This study was funded by the Slovak Scientific Grant Agency under grants VEGA 1/0044/18 and VEGA 1/0190/20 and Ministry of Health of the Slovak Republic under the project registration number 2018/20-UKMT-16.

